# Ambient-Dried Silica Aerogel Powders Derived from Coal Gangue by Using One-Pot Method

**DOI:** 10.3390/ma15041454

**Published:** 2022-02-15

**Authors:** Jian Wei, Pinghua Zhu, Hao Sun

**Affiliations:** 1 Audit Office, Changzhou University, Changzhou 213164, China; wj21089@163.com; 2 Department of Civil Engineering, Changzhou University, Changzhou 213164, China; 15189783093@163.com

**Keywords:** silica aerogel powder, coal gangue, one-pot, ambient pressure drying

## Abstract

In this paper, we report a new and convenient method for the synthesis of insulating aerogel by recycling solid waste coal gangue, which can reduce the industrial production cost of silica aerogels and realize high value-added utilization of solid waste. Sodium silicate was prepared from a cheap industrial waste coal gangue as the precursor for silica aerogels, which was used for silica wet gel preparation by a one pot method; this method of solvent exchange/surface modification was carried out quickly by mechanical stirring process, and the wet gels derived from coal gangue were dried under ambient pressure condition. A high surface area (~748 m^2^/g) nanostructured aerogel with a 3D open porous microstructure was synthesized, which exhibits a low density (~0.18 g/cm^3^) and a superior thermal insulation performance (~0.033 W·m^−1^·K^−1^). More significantly, the synthetic yield of silica aerogel powder by recycling coal gangue can reach 92%.

## 1. Introduction

Silica aerogel is a non-crystalline material formed by nanoparticle aggregation and air as a dispersing medium, which has excellent properties such as low density, low thermal conductivity, large specific surface area and high porosity [1,2,3,4,5]. These features give it great potential for application in the fields of heat science, optics and catalysts. So far, some routes have been developed to synthesize silica aerogels, for example, using tetraethylorthosilicate (TEOS) or tetramethylorthosilicate (TMOS) as a silicon precursor and the supercritical fluid extraction technique for the drying of the gels, which can synthesize excellent aerogels with high porosity [6,7]. However, these routes for synthesizing aerogels have some issues, such as complicated processing, high cost and safety problems. Despite the development of ambient pressure drying, expensive precursor materials and complex surface chemical modification processes still limit its large-scale industrial production [8,9]. In order to dispose of these disadvantages, it is clear that the gels must be synthesized by a cheaper silicon precursor, modified by a simplified modification process and dried under ambient pressure.

Coal gangue is a kind of low carbon, black or grey rock associated with coal seams and discharged during coal mining and washing, rich in silica and alumina. According to the difference of geological conditions, the discharge of coal gangue is 10–15% of raw coal output. Large area stacking of coal gangue not only occupies much land, but also will generate dust and spontaneous combustion, harmful gases such as SO_2_ and NO_x_ will be released to pollute the atmosphere and toxic heavy metals in coal gangue will also contaminate water and soil as rain washes [10,11]. Many application methods of coal gangue have been developed to improve its added value [12]. However, the utilization of coal gangue is still remains below 15%, and the comprehensive utilization of such waste is mainly focused on its application as a building material, such as a concrete filling material and composite cements. Therefore, the development of a new and effective method is beneficial to expand its potential application field in coal gangue resource recovery.

In the recent studies, coal gangue has been used as a cheap silicon precursor to synthesize silica materials, as it is rich in silicon; some studies have been reported to confirm the feasibility of this route. J. Zhu et al. [13] used coal gangue as raw material to synthesize hydrophobic SiO_2_ aerogel; high porosity (88.97%) and a low density (0.256 g/cm^3^) were achieved. L. Dong et al. [14] extracted SiO_2_ using acid leaching and discussed the influencing factors of SiO_2_ extraction, such as leaching time, acid material ratio and leaching temperature. A yield of 68.04% for SiO_2_ can be achieved by this method. P. Zhu et al. [15] offered an environmental route to synthesize insulating aerogel material by recycling solid waste coal gangue; the SiO_2_ aerogel prepared by this route exhibits a typical 3D open porous microstructure with high surface area (690 m^2^/g) and superior thermal insulation performance (0.0265 W·m^−1^·K^−1^). Overall, there are a few reports on the preparation of silica aerogels from coal gangue, but no detailed analysis and systematic characterization of such materials have been performed. The main challenges of aerogel preparation are the control of the high SiO_2_/Na_2_O molar ratio in the leaching sol (about 3:1, requires increasing silica concentration or restricting NaOH loading) [16] and high leaching efficiency of silica (requires a large quantity of solvent and high NaOH concentration). In this case, if we add other silica resources to extract silica out and adjust the SiO_2_/Na_2_O ratio efficiently, both silicon extraction efficiency and SiO_2_/Na_2_O ratio can be ameliorated concomitantly [17,18]. The development of such a feasible route for the preparation of high-performance materials has been motivated, such as silica aerogels and its corresponding composite materials can prepared by recycling this low-cost and environmental correlated large stocked solid waste.

In this work, a process for preparing silica aerogel powder from a high-silicon coal gangue was investigated. Sodium silicate was prepared from coal gangue as the precursor for silica aerogels, and solvent exchange/surface modification process can be rapidly carried out by using a one pot method. The silica surface can be modified using hexamethyldisilazane (HMDS) and heptane for successful ambient pressure drying. The surface of silica was dried successfully under normal pressure and the energy consumption was reduced. A high surface area (~748 m^2^/g) nanostructured powdery aerogel with a 3D open porous microstructure was synthesized in this study, which shows a low density (~0.18 g/m^3^), a decent thermal conductance of 0.033 W·m^−1^·K^−1^ and yield of silica aerogel powder of 92%. In addition, silicon can be effectively extracted from coal gangue by similar processes to prepare nanomaterials such as silica, SiO_2_ aerogel and SiO_2_-Al_2_O_3_ aerogel to achieve high utilization of coal gangue. The study provides a cost-effective route to synthesize silica aerogel powder from recycled solid waste materials, which reduces the production cost of aerogels and realizes the high value-added utilization of coal gangue waste.

## 2. Experimental

### 2.1. Coal Gangue Sample

**Figure 1:**
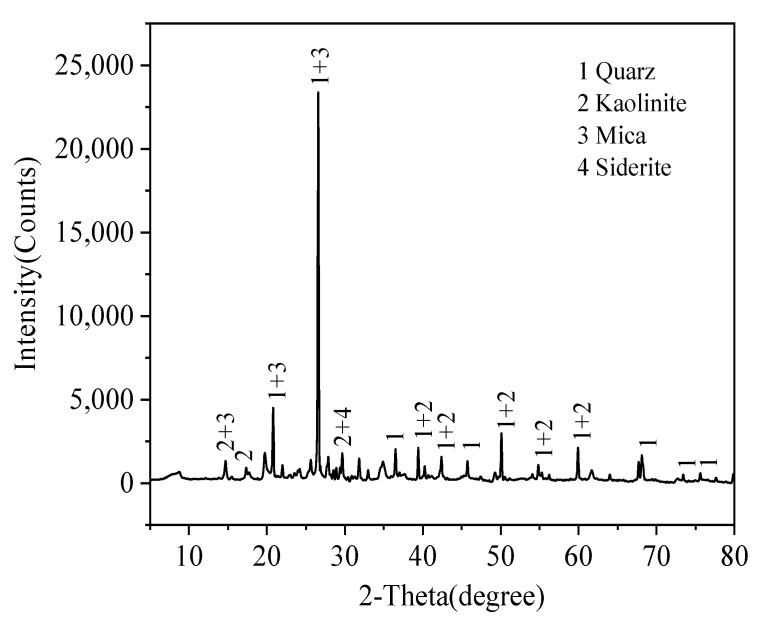
XRD pattern of coal gangue materials.

Jintan Coal Mining Co., Ltd., Changzhou, China, provided raw coal gangue. The raw coal was ground and sieved to less than 300 μm, the sieved coal gangue were dried for 8 h at 105 °C and stored in a desiccator as the coal sample. The content of C in coal sample was less than 15% wt.; Table 1 and Figure 1 show the elemental and mineral analysis results of coal gangue samples by X-ray fluorescence (XRF) and powder X-ray diffraction (XRD), respectively. It can be identified in Table 1 that the contents of Si and Al in the raw coal gangue are high. Figure 1 shows that the main crystalline components of coal gangue are quartz (SiO_2_), kaolinite (Al_2_Si_2_O_5_(OH)_4_) and mica (X_2_Y_4_–_6_Z_8_O_20_(OH)_4_) [19]. Due to the low reactivity of mineral crystals, in order to fully extract silicon from raw materials, further treatment is needed to remove impurities such as aluminum and iron.

### 2.2. Chemicals

Sulfuric acid (H_2_SO_4_, 98%), nitric acid (HNO_3_, 65%) and anhydrous ethanol (EtOH) used in the study were produced by Guoyao Chemical Co., Ltd., China; heptane, hexamethyldisilazane (HMDS), sodium hydroxide (NaOH) and commercial sodium carbonate (Na_2_CO_3_) were produced by Cancheng Chemical Co., Ltd., Shanghai, China. All of chemicals are analytical reagent grade without any further purification.

### 2.3. Method

**Figure 2:**
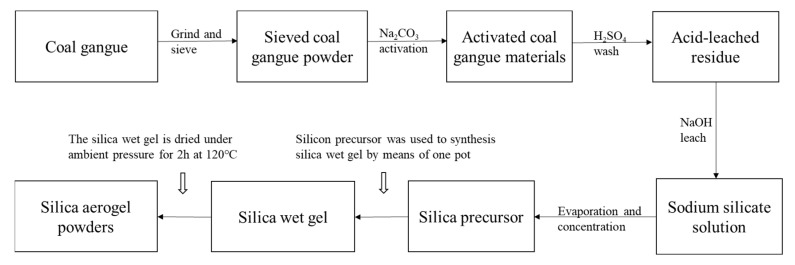
The specific preparation procedure of SiO_2_ aerogel powders by recycling coal gangue.

**Figure 3:**
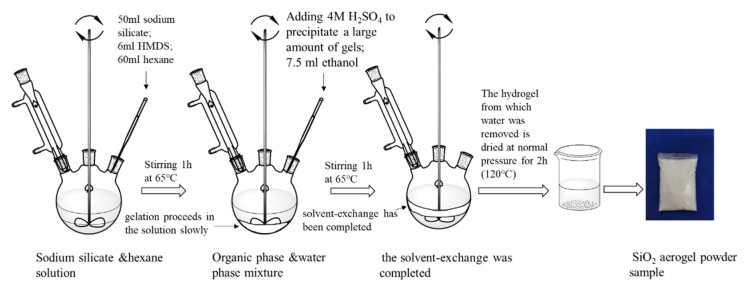
Sodium silicate was used as silicon precursor to synthesis SiO_2_ aerogel powder by means of the one pot method.

The specific preparation procedure of SiO_2_ aerogel powder by recycling coal gangue is shown in Figure 2. Firstly, as-sieved coal gangue samples were mixed uniformity with Na_2_CO_3_ in a mass ratio of 0.6 and calcined at 815 °C (±10 °C) for 2.5 h. Subsequently, the sinter was ground and sieved to less than 300 μm, which sieved sinter was blended with an aqueous solution of H_2_SO_4_ (6 mol/L) and stirred for 2.5 h at 65 °C, where the ratio of sinter/H_2_SO_4_ was 1 g/20 mL. After that, filtration was used to promote the solid–liquid separation. In the process of filtration, the solids were washed with deionized water to ensure that no acid remained. The solid was acid-leached residue from coal gangue after drying for 8 h at 105 °C. The acid-leached residue was blended with an aqueous solution of NaOH (0.5 mol/L) and stirred for 90 min at 90 °C, where the ratio of acid-leached/NaOH was 1 g/20 mL. After filtering unreacted waste residue and finishing the reaction, the solution was a sodium silicate solution. Controlling the content of SiO_2_ in the sodium silicate solution, it was found to be 4–4.5% through evaporation and concentration; titration (GBT 4209-2008) was used to measure the content of SiO_2_ several times in the process. Sodium silicate was used as silicon precursor to synthesis SiO_2_ aerogel powder by means of the one pot method (Figure 3), while the 50 mL of sodium silicate was stirred at 300 rpm, 6 mL of hexamethyldisilazane (HMDS) and 60 mL heptane were added to the solution. After stirring for 1 h, H_2_SO_4_ (4 mol/L) and 7.5 mL ethanol were added slowly and stirred at 400 rpm; gelation slowly proceeded in the solution, and simultaneously solvent-exchange by the organic solvent proceeded in the hydrogel. Subsequently, when the solvent-exchange was completed (within 1 h), the hydrogel from which water was removed was dried under ambient pressure for 2 h at 120 °C to obtain the final silica aerogel powders. The amount of each component required for the preparation of SiO_2_ is shown in Figure 3.

### 2.4. Calculation and Characterization

Mineral and Elemental Analysis. X-ray diffraction (XRD) analysis was used for the qualitative and quantitative analysis of crystalline substances, which were analyzed on an APPEX II DUO X-ray system at 40 mA and 40 kV. The data were collected from a 2-theta degree ranging from 10° to 80° at a step of 6°/min. X-ray fluorescence (XRF) was used for the element analysis, which was analyzed on EA2400II system at 40 mA and 50 kV.

Fourier Transform Infrared Spectroscopy (FT-IR) Characterization. The samples were pretreated by the compression method (KBr = 1:200) and dried completely under the infrared light. The molecular structure of aerogel powders obtained from KBr pellets were analyzed by FT-IR with a Nicolet iS50FT spectrophotometer at the range 4000–400 cm^−1^ and 4 cm^−1^ resolution.

Scanning Electron Microscope (SEM) Characterization. The micromorphology of silica aerogel powder was analyzed by coating them with a 10 nm thick platinum layer. All aerogels were analyzed by SEM on JEOLJSM-6460 LVSEM with 10 kV accelerating voltage and 5 mm working distance.

Brunauer–Emmett–Teller (BET) Specific Surface Area. The Brunauer–Emmett–Teller (BET) method was used to calculate the specific surface area of aerogel powers. The nitrogen adsorption and desorption isotherms of degassed samples at −196 °C were measured on a Micromeritics ASAP 2010C instrument, which were used to analyze the pore structure of aerogel powers. The Barrett–Joyner–Halender (BJH) model was used to obtain the pore size distribution from the desorption branch of the isothermal line. Formulas for calculating pore volume (Vpore) and average pore size (Dpore) are shown as follows:

(1) $$V_{\mathrm{pore}}=\frac{1}{\rho }\;-\;\frac{1}{\rho_{\mathrm{skeleton}}}$$

(2) $$D_{\mathrm{pore}}=\frac{{4V}_{\mathrm{pore}}}{S_{\mathrm{BET}}}$$

where ρ is the density of the aerogel powder, ρ_skeleton_ is the density of aerogel powder skeleton, and the S_BET_ is the specific surface area of aerogel powder using BET.

Thermal Conductivity. Measurement of thermal conductivity of the aerogel powder materials by an in-house built transient hot-wire device. The Cu/Ni alloy wire was chosen as 73 mm in length and 0.127 mm in diameter to obtain the optimum ratio of 575. The thermal conductivity was calculated as follows [20]:

(3) $$\lambda =\frac{\mathrm{VI}/4\pi L}{\mathrm{dT}/d(\mathrm{lnt})}$$

where V and I are the voltage and current, fixed at 0.3 V and 0.09 A in this experiment; L means the length of Cu/Ni alloy wire (73 mm,0.15 Ωmm^2^/m) and dT/d(lnt) represents the average fitting slope of the measurements.

Extraction Yield of Aerogel Powders derived from Coal gangue. The extraction yield is the ratio of the actual output of products to the theoretical output. In this study, the actual production is the mass of aerogel powders and the theoretical output is the content of SiO_2_ in a certain mass of coal gangue. The yield was calculated as follows:

(4) $$\partial =\frac{m}{m_{1}\cdot \omega_{1}{\;-\;m}_{2}\cdot \omega_{2}}\times 100\%$$

where m represents the mass of silica aerogel powders derived from m_1_ coal gangue (g), m_1_ represents the mass of raw coal gangue (g), w_1_ is the content of SiO_2_ in the raw coal gangue (%), m_2_ represents the mass of unreacted waste residue after NaOH leach (g) and w_2_ is the content of SiO_2_ in the unreacted waste residue (%).

## 3. Results and Discussion

### 3.1. The SiO_2_ Extraction from Coal Gangue

In order to extract relatively pure silica from coal gangue for the fabrication of silica aerogel materials, most of soluble impurities could be removed by calcination and acid leaching firstly. Mixed coal gangue and Na_2_CO_3_, the mixture was calcined at 815 °C to transfer stable silicon-rich compounds, such as kaolinite and quartz. Calcined sinters were blended with an aqueous solution of H_2_SO_4_ to leach out impurities, such as Fe^2+^, Al^3+^, K^+^ and SO_3_. It is thought that the major reaction routes are as follows [21]:

Fe_2_CO_3_ + Na_2_CO_3_ + O_2_ → Na_2_O·Fe_2_O_3_ + CO_2_ (5)

Na_2_CO_3_ + Al_2_O_3_ → 2NaAlO_2_ + CO_2_ (6)

Na_2_CO_3_ + 2C → 2Na + 3CO (7)

2Na + CO_2_ → Na_2_O + CO (8)

3Na_2_O + 6SiO_2_ + 3Al_2_O_3_ → Na_6_(AlSiO_4_)6 (9)

Na_6_(AlSiO_4_)_6_ + 24H^+^ → 6Na^+^ + 6SiO_2_ + 6Al^3+^ + 12H_2_O (10)

**Figure 4:**
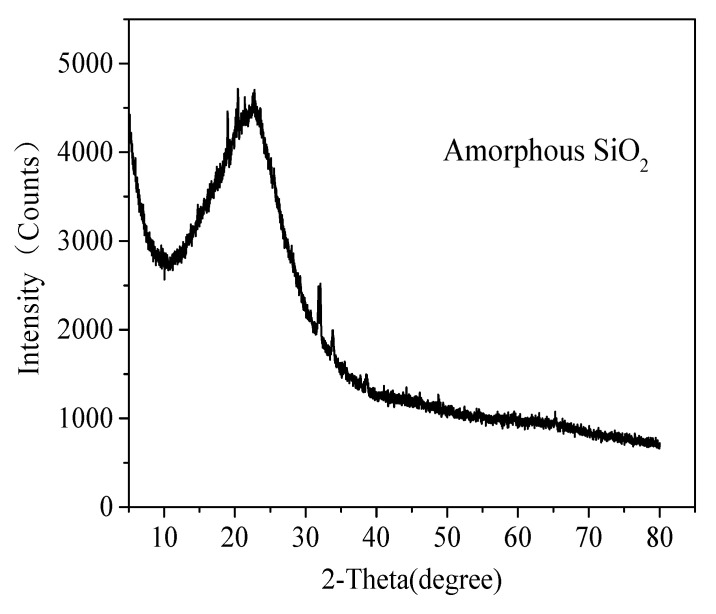
XRD pattern of acid-leached residue.

After calcining with Na_2_CO_3_ and H_2_SO_4_ leaching, most of the impurities, such as Fe_2_CO_3_ and Al_2_O_3_, are almost completely removed. Table 2 and Figure 4 shows the XRF and XRD analysis of acid-leached residue from coal gangue, respectively. It can be seen from XRF that samples showed a relatively high SiO_2_ contents, over 95 wt%. The XRD pattern indicates an effective removal of impurities from coal gangue and most of silicon phases were still retained, the acid-leached residue from coal gangue pretreated by this new route is amorphous without any diffraction peaks for crystalline silica. Amorphous silica is more easily dissolved in NaOH solution to form sodium silicate (Na_2_SiO_3_), the usual and cheap precursor for silica aerogels [22,23].

### 3.2. Characteristics of the SiO_2_ Aerogel Powders

The physical properties of the silica aerogel powders derived from coal gangue by means of one pot are summarized in Table 3. Aerogel particles were prepared by the sol-gel method and dried at normal pressure, using tetraethyl orthosilicate (TEOS) as silicon source and NH_4_F as catalyst. Commercial silica aerogels were purchased from Guangdong Alex Co., Ltd., Guangdong, China. It can be seen from Table 3 that the physical properties of aerogel powders are similar to those of aerogel particles and commercial aerogels, which proves the feasibility of this new route to synthesize silica aerogels [24,25,26].

**Figure 5:**
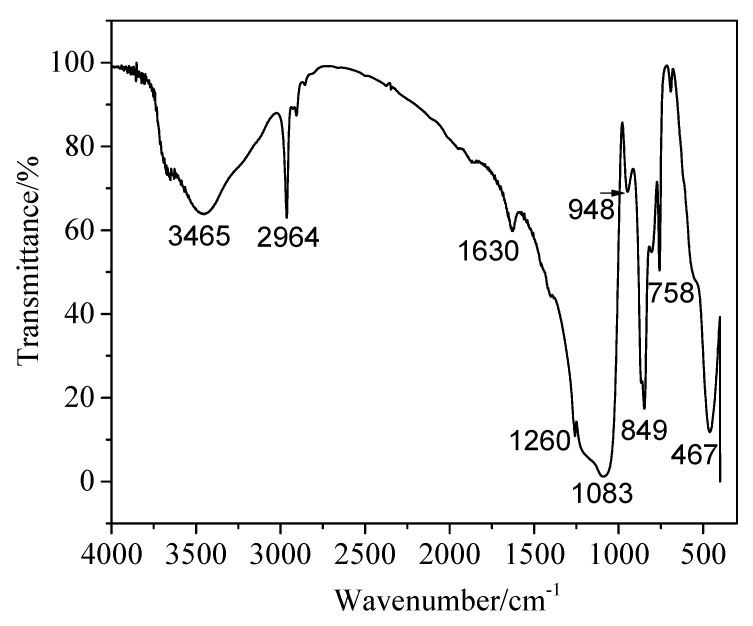
FT-IR spectra of SiO_2_ aerogel powders.

Figure 5 is the infrared absorption spectrum (FT-IR) after modification of silica aerogel powders. The broad peaks at about 3465 cm^−1^ and 1630 cm^−1^ show a weaker absorption intensity; they are -OH groups due to the H_2_O absorbed through KBr during FTIR test. Obviously, the peaks of aerogel powder at 1083 cm^−1^ and 467 cm^−1^ have a complete silica network, corresponding to the asymmetric stretching vibration of Si-O-Si bonds [27]. The peaks at about 849 cm^−1^ and 2964 cm^−1^ are attributed to the existence of a Si-C bond and the vibration of a C-H bond, respectively. The presence of C–H bonds and Si–C bonds reveals that silica aerogels have been successfully modified by the CH_3_ groups [28]. Silica aerogel powders are hydrophobic after surface modification prepared by means of one pot. Hydrophobicity is determined by the degree to which -Si-CH_3_ replaces -OH on the silicon surface; it can be seen from FT-IR pattern that a certain amount of Si-OH groups were decreased and -CH_3_ groups from HMDS were attached to the gel surface. The peak at 1260 cm^−1^ is ascribed to Si-CH_3_ bonds and the peak at 948 cm^−1^ is ascribed to Si-OH bonds [29].

**Figure 6:**
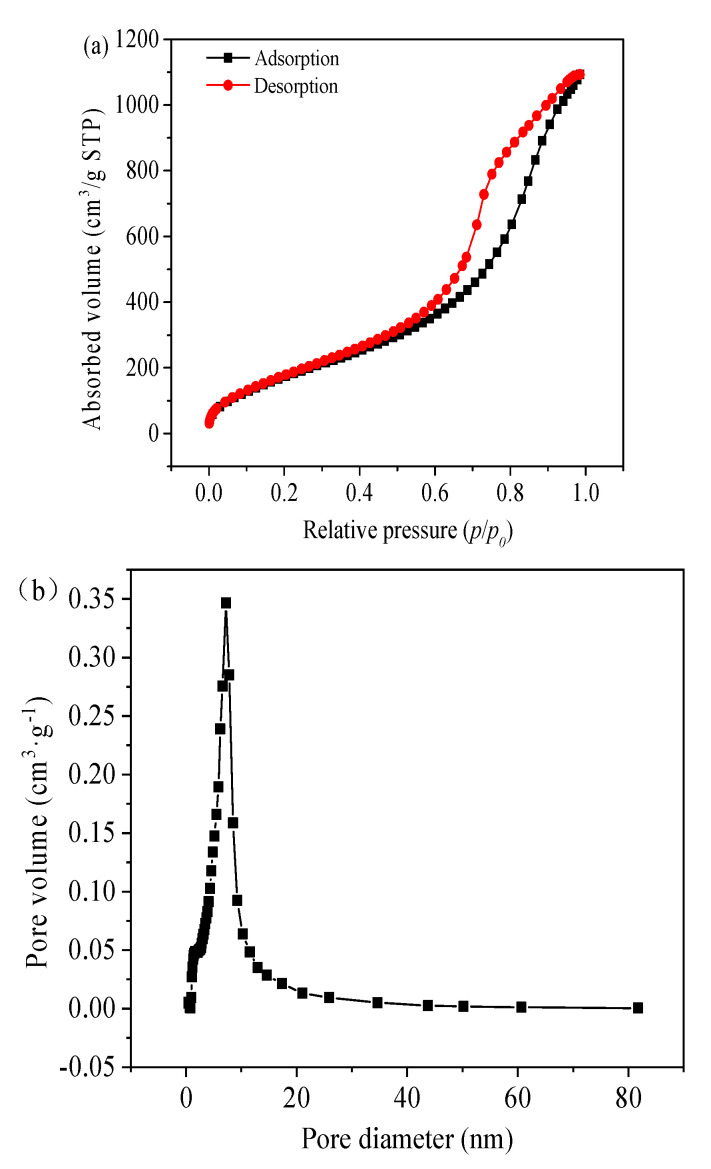
N_2_ adsorption-desorption of the silica aerogel powders: (**a**) BET isotherm; (**b**) BJH pore size distribution (PSD) derived from desorption branch.

The relevant pore size distribution and N_2_ adsorption–desorption isotherms of the silica aerogel powders are shown in Figure 6. The isotherm of this aerogel powder is close to a type IV; the adsorption branch slowly rises in the relatively small pressure region, which is monolayer adsorption. Most of the absorption occurs between relative pressures of 0.1 and 0.95, indicating a typical mesoporous structure. The specific surface area, pore size and pore volume of T aerogel are 748 m^2^/g, 7.04 nm and 1.64 cm^3^/g, respectively. The analysis of the pores was conducted by using BJH equilibrium model. Most of the pore sizes of aerogels ranged from 2 nm to 80 nm, which indicated that the aerogels prepared by this method were typical nanoscale mesoporous materials. Silica aerogel powders are quite similar to a normal silica aerogel and should present identical performance, such as thermal insulation [30].

**Figure 7:**
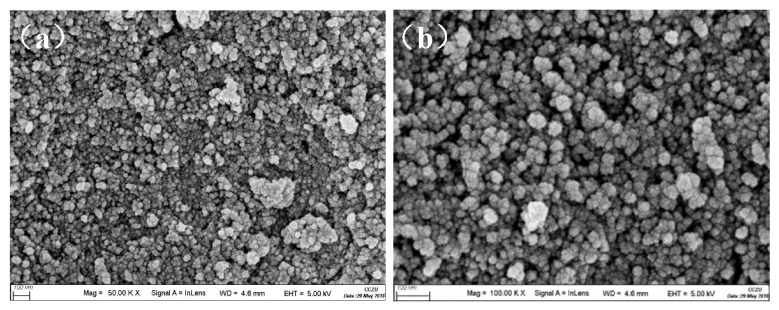
SEM image of silica aerogel powders at (**a**) 50.00 KX and (**b**) 100.00 KX magnification.

The SEM (Figure 7) of the silica aerogel powders prepared by this route exhibits a typical mesoporous three-dimensional network structure. As can be seen from the image, the sample mainly consists of nanoparticles and the size is approximately less than 50 nm. The aerogels powders have good uniformity, uniform pore distribution and a loose structure, which is a kind of porous nano-mesoporous material with continuous network structure.

**Figure 8:**
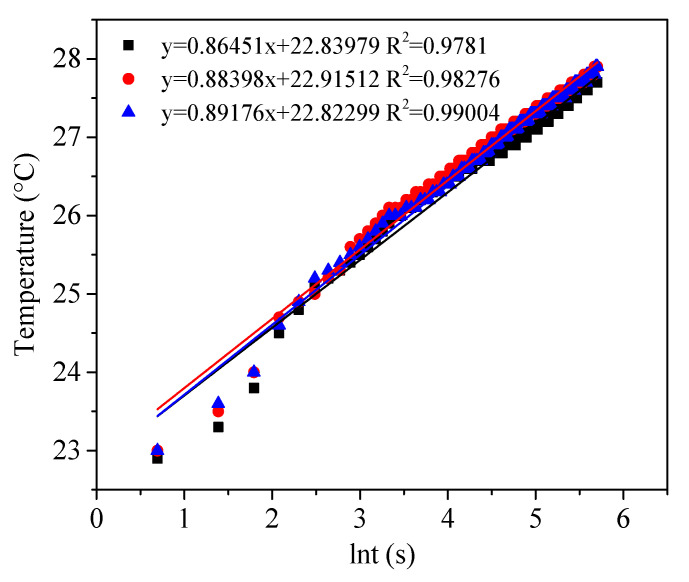
Evaluation of thermal conductivities of aerogel powder by transient hot-wire method.

One of the main applications of silica aerogel materials is thermal insulation. The thermal conductivity of the aerogel granulate was measured by the transient hot-wire method, which was recorded every 2 s and calculated from (3) by using the slops of Figure 8. A thermal conductivity of 0.033 W·m^−1^·K^−1^ was achieved.

## 4. Conclusions

In this study, SiO_2_ aerogel powders were prepared by recycling solid waste coal gangue using a one pot method. Low density (0.18 g/cm^3^), high specific surface area (above 700 m^2^/g), high porosity (above 90%) and good insulation (0.033 W·m^−1^·K^−1^) were displayed by the aerogel powder prepared by the route provided in this paper, which are consistent with the aerogel prepared by a typical TEOS precursor that are reported in literature. The preparation of SiO_2_ aerogels can be used to adsorb pollutants in water and harmful gases in air, and related tests need to be further improved. In addition, the dosages of ethanol, HMDS and H_2_SO_4_ were adjusted according to the experimental phenomena, and the effects of various parameters on the structure and properties of the prepared aerogels need to be comprehensively analyzed.

This work exhibits a feasible and economical route to thermal insulating silica aerogel powders from recycled coal gangue solid waste, which offers insights into a cost reduction and a process simplification process for the industrial production of silica aerogel powders.

## Figures and Tables

**Table 1:** Chemical composition analysis of the raw coal gangue (wt%).

SiO_2_	Al_2_O_3_	CaO	SO_3_	Fe_2_O_3_	K_2_O	MgO	Na_2_O	TiO_2_	P_2_O_5_	V_2_O_5_	Total
44.93	17.84	14.04	11.43	4.24	2.62	2.03	1.99	0.46	0.28	0.14	100

**Table 2:** Chemical composition analysis of the acid-leached residue (wt%).

SiO_2_	Na_2_O	Al_2_O_3_	SO_3_	Fe_2_O_3_	TiO_2_	CaO	K_2_O	MoO_3_	Total
95.9	1.2	1.01	0.73	0.38	0.29	0.2	0.19	0.1	100

**Table 3:** The physical properties of several aerogel products.

Sample Identification	Density (g/cm^3^)	BET Surface Area (m^2^/g)	Thermal Conductivity (W·m^−1^·K^−1^)	Porosity (%)
Aerogel powders	0.18	above 700	0.033	above 90
Aerogel particles	0.19	above 650	0.031	above 90
Commercial aerogels	0.018	500–650	0.013	95–98
